# A Comparative Analysis of Bioactive Compounds and Heavy Metal Content in Table Eggs from Various Housing Systems

**DOI:** 10.3390/foods15162835

**Published:** 2026-08-14

**Authors:** Kamil Drabik, Renata Zdun, Piotr Skałecki, Ewa Januś, Hanna Spasowska, Justyna Batkowska

**Affiliations:** 1 Institute of Biological Basis of Animal Production, University of Life Sciences in Lublin, 13 Akademicka St., 20-950 Lublin, Poland; kamil.drabik@up.edu.pl (K.D.); zdun.renata.anna@gmail.com (R.Z.); 2 Department of Quality Assessment and Processing of Animal Product, University of Life Sciences in Lublin, 13 Akademicka St., 20-950 Lublin, Poland; piotr.skalecki@up.edu.pl; 3 Department of Cattle Breeding and Genetic Resources Conservation, Faculty of Animal Sciences and Bioeconomy, University of Life Sciences in Lublin, 13 Akademicka St., 20-950 Lublin, Poland; ewa.janus@up.edu.pl; 4 Department of Administrative Law and Administrative Science, Institute of Legal Sciences, Maria Curie-Sklodowska University, 5 Maria Curie Sklodowska Sq., 20-031 Lublin, Poland; hanna.spasowska@mail.umcs.pl

**Keywords:** bioactive compounds, heavy metals, fatty acid profile, fat-soluble vitamins, food safety, organic farming

## Abstract

The growing demand for eggs from alternative housing systems is driven by perceived nutritional and welfare benefits. However, the trade-off between enhanced bioactive composition and exposure to environmental contaminants remains insufficiently explored. This study evaluated the physical traits, bioactive compounds, and heavy metal content of 480 commercially available table eggs from four housing systems: organic, free-range, barn, and enriched cages. Organic eggs exhibited the highest albumen quality, expressed by Haugh units (92.03 vs. 70.86 in cages) and lysozyme activity (144,877 U/mL vs. 119,830 U/mL in cages), alongside the highest concentrations of fat-soluble vitamins (e.g., vitamin A at 1600 μg/100 g and vitamin E at 3267 μg/100 g) and n-3 polyunsaturated fatty acids, including docosahexaenoic acid (DHA: 0.673% vs. 0.360% in cages). Conversely, organic eggs presented the weakest eggshells (breaking strength of 34.19 N vs. 47.05 N in cages) and a less favourable lipid profile regarding atherogenicity (AI: 0.411) and thrombogenicity (TI: 3.455) indices. Crucially, organic eggs contained significantly higher levels of lead (0.361 mg/kg) compared to indoor systems (0.041 mg/kg in barn and 0.102 mg/kg in cages), highlighting their susceptibility to environmental pollution. Cage and barn eggs were associated with higher chemical safety and shell strength but demonstrated a lower overall bioactive value. Ultimately, the choice of table eggs may involve a trade-off between the health-promoting benefits of extensive systems and the potential risks of heavy metal contamination, emphasising the need for integrated food safety management.

## 1. Introduction

Chicken eggs are among the most significant and cost-effective sources of high-quality animal protein globally. Owing to their affordability and the absence of legislative or religious dietary restrictions, they constitute one of the most widely utilised animal-derived ingredients worldwide, particularly in developing countries [1]. Beyond their protein content, eggs provide significant quantities of polyunsaturated fatty acids [2], almost all essential vitamins (excluding vitamin C) [3] and minerals. Given their substantial consumption and high nutritional density, eggs are well-suited as a matrix for developing functional foods aimed at mitigating nutritional deficiencies in human populations. Previous initiatives have successfully demonstrated the biofortification of eggs with vitamin D (e.g., the Sunshine Eggs Project^®^) [4] and essential minerals [5].

The chemical composition and overall quality of eggs are strongly influenced by factors related to the laying hen’s genotype, nutritional regimen, and housing system. In the context of laying hen management within the EU, the primary regulatory legislation is Council Directive 1999/74/EC of 19 July 1999 [6], which mandated the housing of birds in enriched cages and established the fundamental welfare requirements for non-cage (alternative) rearing systems. In commercial practice, this classification was further specified by Directive 2002/4/EC [7] on the registration of establishments keeping laying hens, which explicitly requires the use of the consumer-recognised labelling scheme for identifying the housing method from which the eggs originate. From a legislative perspective, significant amendments concerning table eggs on the market were also introduced by Commission Implementing Regulation (EU) 2023/2466 [8], which details the marketing standards for eggs and mandates their proper stamping to ensure full traceability [9].

As with most commercial food products, table eggs must not only comply with rigorous quality standards but also meet consumer expectations. While consumer choices exhibit significant regional variation, there has been a marked increase in demand for eggs derived from alternative (non-cage) housing systems in recent years. Although statistical data indicate that the majority of eggs produced both within the EU [10] and globally [11] still originate from cage systems, mounting social pressure has prompted several retail chains to eliminate cage-sourced eggs from their supply chains. This phenomenon, referred to in the literature as market displacement, renders the production of eggs stamped with code 3 economically vulnerable due to steadily shrinking distribution channels [12]. Even though overarching EU legislation currently permits the use of enriched cages, the legal landscape across Europe is experiencing dynamic polarisation. Utilising their prerogative to enforce stricter animal welfare standards than the EU baseline, certain Member States have introduced total bans on cage housing. Luxembourg and Austria pioneered this movement (with the latter banning enriched cages in 2020), and Germany followed suit in 2025 [13]. This legislative transformation is also evident in Central Europe, exemplified by Czechia, which has enacted a comprehensive ban on all cage systems effective from 2027 [14].

The housing system can directly influence egg quality, primarily due to inherent differences such as access to outdoor runs [15]. Previous research has predominantly focused on the traits of specific morphological components of the egg. Studies have demonstrated the effect of the housing system on various parameters, including egg weight [16,17], shell quality and cleanliness [18,19], and albumen quality traits [20]. Particular attention has also been given to yolk colour, which appears to be determined by the birds’ access to green forage rich in plant pigments [21].

Although consumers generally believe that alternative (non-cage) systems not only enhance laying hen welfare but also positively affect the quality of the eggs produced, including a higher concentration of desirable health-promoting substances such as fat-soluble vitamins and a more favourable fatty acid profile, it should be recognised that as the degree of production extensification increases, the level of control over zoohygienic conditions and the forage consumed by the birds in outdoor runs consequently decreases. In this context, studies indicate a higher prevalence of microbiological contamination on the eggshells from free-range systems [22,23]. Furthermore, access to outdoor runs and the opportunity to consume green forage inevitably lead to a partial loss of producer control over the overall quality and safety of the birds’ diet. Our previous research in this area [24] demonstrated that despite the stringent regulations governing organic farming, it is impossible to fully control the heavy metal concentrations in eggs originating from this housing system. This limitation arises from unavoidable environmental factors, such as the airborne transport of metallic pollutants via particulate matter and their subsequent deposition onto the surface of the outdoor runs.

This issue is further compounded from a consumer perspective. Typically, consumers select table eggs based on easily observable or economic criteria such as freshness, price, housing system labels, shell colour, structural integrity (absence of cracks), and occasionally brand familiarity. However, they lack direct influence over the actual quality of the eggs available on the market, particularly regarding their detailed chemical composition and the potential presence of heavy metals. Furthermore, the average consumer possesses limited genuine understanding of how different housing systems, actual bird welfare, and specific dietary regimens ultimately dictate the nutritional profile and safety of the final product. At the same time, avian eggs are increasingly recognised not merely as basic commodities, but as complex sources of bioactive components, where specific albumen-derived proteins and peptides exhibit remarkable immunomodulatory and health-maintaining properties [25]. However, recent studies emphasise that modern evaluation of egg quality must comprehensively account for how rearing environments, technological parameters, and biochemical profiles interact within the final food matrix [26]. Consequently, there is a paucity of scientific literature that simultaneously and comprehensively contrasts the benefits derived from an elevated nutrient content against the risks associated with chemical environmental contaminants within the same food matrices. While previous studies have explored individual parameters of poultry production, the complex, multidimensional relationships among housing conditions, diet, overall egg composition, specific albumen proteins, and environmental contaminant exposure remain largely unresolved.

Considering the aforementioned factors, it is essential to balance the benefits and potential hazards associated with various rearing systems, specifically regarding egg quality, chemical composition, and heavy metal contamination. The main objective of this study was to conduct a comprehensive evaluation of the physicochemical quality and bioactive compound content (including fatty acids, vitamins, and minerals) of table eggs, in relation to the housing system. The research focuses on a risk–benefit analysis, illustrating the inherent trade-off between the health-promoting characteristics of eggs from organic and free-range systems and the potential threat posed by elevated levels of heavy metal contamination.

## 2. Materials and Methods

### 2.1. Experimental Design and Egg Collection

The present study was conducted within the comprehensive research framework of the “Regional Initiative of Excellence” project (Grant No. 029/RID/2018/19). The overarching objective of this initiative was to evaluate the bioactive composition of animal-derived foods directly available on the retail market. By doing so, the project aimed to empirically verify prevailing consumer perceptions regarding the health benefits of alternative production systems and to assess the risk–benefit balance between a rich bioactive profile and potential toxicological threats. In alignment with these broader goals, this specific study was designed as an observational quasi-experiment. This approach was deliberately chosen to accurately reflect real-world commercial poultry production scenarios, where controlling all environmental variables is neither feasible nor representative of the actual consumer market. Consequently, the inherent variability of alternative housing systems, such as weather fluctuations, soil parameters, and access to natural forage, was treated as a natural component of the production environment rather than a confounding factor. Regarding ethical considerations, the approval of an Institutional Animal Care and Use Committee was not required. The study did not involve any direct in vivo interventions, physiological manipulations, or harm to live animals; all analysed samples, comprising table eggs and corresponding feed batches, were acquired directly through commercial distribution channels, thereby accurately mimicking standard consumer purchasing practices.

A total of 480 fresh, grade A, table eggs were purchased from a single packing station in the Lublin Voivodeship (Poland). This observational approach was intentionally designed to reflect systemic, commercial-level product quality corresponding directly to the legal definitions of housing systems as encountered by consumers on the retail market. The eggs originated from four different housing systems (120 eggs per group): organic (code 0), free-range (code 1), barn/floor (code 2), and enriched cages (code 3). The specific suppliers delivering these egg batches to the packing facility were requested to provide corresponding samples of the layer diets used during the collection period. Notably, both the free-range and barn eggs originated from the same supplier; consequently, the hens in both of these housing systems were fed an identical commercial feed mixture.

### 2.2. Physical Egg Quality Traits

Egg quality analyses were performed within 48 h of collection. Therefore, while post-lay age naturally influences internal parameters, all groups were evaluated under identical, legally defined commercial standards representative of retail availability (the use-by date was exactly 21 days in each case). The assessment of both external and internal egg quality defects was performed according to the methodology described by Wengerska et al. [27]. This classification system enabled the systematic identification and quantification of specific shell defects (e.g., severe stripe marks, points, pimples) and internal quality abnormalities (e.g., air cell mobility, chalazae condition). The egg quality evaluation included:

1. Whole egg traits:
  • Egg weight was measured using an electronic scale (±0.1 g);
  • Specific gravity (density, g/cm^3^) was determined based on Archimedes’ principle using displacement in water and air;
  • The shape index was calculated as the percentage ratio of width (short axis) to length (long axis) using a digital calliper;
  • The percentage share of egg components (yolk, albumen, and shell) was calculated relative to the total egg weight.

2. Shell traits:
  • The strength (N) was determined using an Instron Mini 55 universal testing machine (Instron®, Norwood, MA, USA);
  • Colour (%) was measured with a reflectometer (expressed as percentage of light reflected from 0 to 100);
  • Thickness (mm) was measured at the equator using a EQM micrometer screw (TSS®, York, UK);
  • Density (g/cm^3^) was calculated according to Shafey [28].

3. Albumen and yolk traits:
  • Thick albumen height (mm) was determined on a mirror table using an EQM (Egg Quality Measurement) sensor (TSS®);
  • The Haugh units (HU) were calculated according to Williams [29];
  • The pH of the albumen was measured using a CP-251 pH-metre (Elmetron, Zabrze, Poland);
  • Yolk colour was scored using a colorimeter calibrated against the 16-point Yolk Colour Fan (DSM Nutritional Products, Basel, Switzerland).

### 2.3. Chemical and Bioactive Analysis of Eggs

Yolk samples were pooled by group for subsequent chemical characterisation. Each pooled sample consisted of three yolks from the same group, and a total of 15 pooled samples were analysed (representing 45 eggs in total). These 15 pooled samples were used for the determination of both fatty acid profiles and vitamin content. The fatty acid profile and lipid indices were determined via Gas Chromatography (GC-FID) according to the method described by Batkowska et al. [30]. Based on the content of individual fatty acids and their relative proportions, a number of lipid quality indices were calculated: the susceptibility to peroxidizability index (PI) according to Arakawa and Saga [31], the atherogenic index (AI) and the thrombogenic index (TI) according to Ulbricht and Southgate [32], the content of desirable fatty acids (DFA) according to Medeiros et al. [33], the concentration of hypercholesterolaemic saturated fatty acids (HSFA) according to Renna et al. [34], as well as the ratio of hypo- to hypercholesterolaemic fatty acids (h/H) proposed by Domaradzki et al. [35].

Fat-soluble vitamins (A, D3, and E) were analysed using a High-Performance Liquid Chromatography (HPLC) system equipped with a C18 column and UV-Vis/Fluorescence detectors (Agilent 1260 Infinity II HPLC system, Agilent Technologies, Santa Clara, CA, USA), following the protocol of Mattila et al. [36]. Total cholesterol content was determined according to Kasperek et al. [37]. For mineral composition, yolk samples (*n* = 15) underwent wet microwave digestion and were analysed via atomic absorption spectrometry (AAS) and Inductively Coupled Plasma Mass Spectrometry (ICP-MS) as described previously [24,38]. Lysozyme activity in the albumen was evaluated using 24 individual samples per housing system. The samples were stored at −18 °C, thawed prior to analysis, and enzyme activity was determined spectrophotometrically (according to Wengerska et al. [39]).

### 2.4. Chemical and Toxicological Analysis of Feed

Feed samples provided by the farms were ground to a homogeneous gradation using a laboratory mill IKA M 20 (IKA, Warsaw, Poland). All feed analyses were performed in triplicate and included

• Proximate composition—dry matter, crude fibre, crude ash, and nitrogen content (crude protein) were determined according to standard AOAC [40] methods;
• Heavy metals—cadmium (Cd), arsenic (As), and lead (Pb) concentrations were measured via ICP-MS after acid digestion [24];
• Fatty acid profile—fat extraction and fatty acid methylation were performed prior to GC-FID analysis according to Batkowska et al. [30].

### 2.5. Statistical Analysis

Statistical processing was performed using SPSS 24.0 software [41]. Data normality was verified using the Shapiro–Wilk test. Group means were compared using a one-way analysis of variance (ANOVA) followed by Tukey’s post hoc test for multiple comparisons. For variables that deviated from a normal distribution, the non-parametric χ^2^ (chi-squared) test was applied. Statistical significance was set at *p* ≤ 0.05.

## 3. Results

It should be noted that the table eggs used in this study were acquired through commercial retail channels, which aligned with the project’s objective of simulating real-world consumer choices. Consequently, the researchers had no control over the hens’ genotype or age. Therefore, certain physical egg traits, such as egg weight or shell colour, may have been influenced by these factors rather than solely by the housing system. While parameters such as component proportions, Haugh units, fatty acid profiles, vitamin content, and mineral concentrations strongly reflect the conditions of the housing system and associated management practices, these traits can also be modulated by underlying factors including hen genotype, age, feed composition, storage history, and seasonal variations. Therefore, all findings are presented as associations characteristic of commercial retail availability, reflecting the complex, multifactorial background of table egg production.

To exclude the influence of dietary composition variability on the results, feed samples were collected from each farm supplying the eggs and subjected to detailed analysis. The proximate chemical composition, fatty acid profile, and heavy metal content (Cd, Pb, As) of the feed were determined, with the data presented in the Supplementary Materials (Tables S1–S3).

### 3.1. Physical Egg Quality Traits

Table 1 summarises the frequency of egg and eggshell quality defects according to the hens’ housing system. Statistical verification revealed that the organic system exhibited the highest proportion of defect-free eggs. Conversely, the highest overall frequency of quality defects was recorded in the barn system.

Regarding external eggshell defects, the predominant anomalies identified were those classified in the assessment systems as severe stripe marks and points. Eggs from the cage system exhibited the highest incidence of these changes, resulting in the greatest degree of shell mottling (translucency). Interestingly, wrinkled shells were not observed in the organic egg group, whereas shell marbling was entirely absent in samples obtained from hens kept in the free-range system. Furthermore, eggs from the intensive barn system showed the greatest variation in shape index abnormalities and the highest frequency of structural shell defects, such as weak ends and pimples. Concurrently, the analysis of internal defects revealed specific trends in the organic egg group; in this housing system, a significantly higher frequency of movable air cells and broken chalazae was recorded compared to the remaining groups.

Analysing whole egg quality traits according to the housing system (Table 2), it was observed that eggs with the significantly highest weight were obtained from hens housed in the barn system. Conversely, eggs from the organic production system exhibited the lowest weight. Significant differences related to the housing system were also recorded for the egg shape index. It was observed that eggs from the barn system displayed the most elongated shape, whereas the lowest values for this parameter were recorded for eggs from the cage system. No significant differences were observed in this regard between the organic and free-range system groups.

An important aspect of table egg assessment is the proportion of individual morphological components relative to the total egg weight. The analyses demonstrated that eggs from the cage system were characterised by a significantly higher yolk proportion, whilst the remaining groups did not differ significantly from one another. An opposite trend was observed for albumen proportion. The highest values were found in eggs from the organic system, whereas the lowest values were recorded in the cage system. No significant differences were observed regarding the shell proportion of the total egg weight, regardless of the housing system.

Table 3 presents the shell quality parameters according to the hens’ housing system. The analyses demonstrated that the housing system significantly influenced most of the evaluated physical shell traits. Regarding breaking strength, distinct differences were observed. Shells from the free-range and cage systems exhibited significantly higher breaking strength, while the lowest values for this parameter were recorded in the organic and barn groups.

The housing system also significantly affected shell colour, expressed as a percentage of light reflectance. Eggs from the cage system were characterised by significantly darker shells. Conversely, the lightest shells were observed in the barn system, whereas the organic and free-range systems did not differ significantly from one another. Regarding other parameters, the highest shell weight was recorded in the free-range system. In terms of shell thickness, the highest values were observed in samples obtained from hens kept in the cage system. Eggs from the organic system were characterised by the significantly thinnest shells and lowest weight; however, concerning thickness, these values did not differ from those obtained in the barn system. Analysis of the shell surface area revealed significant differences among all housing systems, with the highest values for this parameter were recorded in the free-range system, and the lowest in the organic system. Notably, the hens’ housing system did not have a statistically significant effect on shell density, the values of which remained uniform across all analysed groups.

Internal egg quality parameters are presented in Table 4. The highest albumen weight was recorded in eggs from the free-range system, whereas the lowest values were observed in the organic group. Regarding the quality assessment of this component, eggs from the organic system exhibited the greatest thick albumen height and, simultaneously, the highest Haugh unit values. The lowest values for both parameters were recorded for samples from hens kept in the cage system. It should be noted that all groups differed significantly from one another in both of these traits. Statistically significant differences were also observed in terms of albumen pH. Eggs from the cage system were characterised by the highest pH value, while the lowest results were obtained for eggs from hens in the free-range system.

Based on the collected data, yolk quality traits were determined for the individual housing systems. Yolk weight was significantly affected by the birds’ housing system. Eggs from the cage system were characterised by a significantly higher yolk weight, while the lowest values were recorded for samples from hens kept under organic conditions. No significant differences were found between eggs from the cage and barn systems. The housing system also significantly modified yolk colour. Eggs from the barn system were characterised by the darkest pigmentation, whereas the lowest values for this parameter were obtained for eggs from the organic system. This observation is particularly interesting, as consumers often perceive a dark yolk colour as one of the key features identifying eggs from high-welfare systems that provide hens with access to outdoor runs. Regarding the occurrence of meat and blood spots within the egg content, meat spots were more frequently observed than blood spots in all analysed housing systems. Their highest incidence was recorded in the cage system, followed by the free-range system. Conversely, the frequency of meat spots in the organic and barn systems remained similar.

### 3.2. Fatty Acids Analysis

The housing system significantly affected the fatty acid profile of egg yolks (Table S4). The organic system showed the greatest number of differences, being characterised by significantly higher levels of most saturated fatty acids (except C17:0), as well as higher contents of C14:1 n-5, C16:1 n-7, C18:3 n-3, and C22:6 n-3 compared to the other systems. In contrast, eggs from the free-range system exhibited the highest values for C17:0 and C20:1 n-9, together with increased concentrations of C18:2 n-6 and C20:2 n-6. The cage system was distinguished by the highest oleic acid (C18:1 n-9) content. No significant differences among housing systems were found for C18:3 n-6, C20:4 n-6, or C20:3 n-3.

Based on the obtained fatty acid profile, the fatty acid indices were also calculated, as presented in Table 5. The analyses revealed a significant influence of the laying hen housing system on most of the determined lipid profile quality indices of egg yolks. The saturated fatty acid (SFA) content was significantly higher in the organic system compared to all other groups, which did not differ from one another. For monounsaturated fatty acids (MUFA), the highest level was found in the cage system, which significantly exceeded the organic and free-range groups, whereas the barn system did not differ significantly from either of them. The total polyunsaturated fatty acid (PUFA) content was significantly higher in the free-range system than in the organic and cage systems, while the barn system occupied an intermediate position. For the sum of n-3 fatty acids, which include α-linolenic acid and DHA, recognised for their beneficial effects on cognitive function and the cardiovascular system, a clearly higher level was observed in the organic system, which significantly exceeded all other groups, while the free-range, barn, and cage systems did not differ significantly from one another. In the case of the sum of n-6 fatty acids, the highest content was found in the free-range system, significantly higher than in the remaining groups, which showed no differences between each other. The content of n-9 fatty acids was significantly higher in the cage system compared to the organic and free-range systems, whereas the barn system did not differ significantly from either of them.

Among the lipid quality indices evaluated, the peroxidability index (PI), which defines the susceptibility of fat to oxidation, showed no significant differences between the systems. In contrast, the atherogenicity index (AI), indicating the pro-atherogenic potential of the diet, was significantly higher in the organic system compared to all other systems, which did not differ significantly from one another. These results indicate a less favourable atherogenic profile of lipids derived from organic eggs. For the Thrombogenicity Index (TI), reflecting the tendency to form blood clots, the highest values were recorded in the organic and cage systems, significantly higher than in the free-range system, while the barn system did not differ significantly from either of them. The Desirable Fatty Acids (DFA) index, defining the share of desirable fatty acids in the lipid profile, was significantly lower in the organic system compared to all other systems, which did not exhibit differences between each other. The content of hypercholesterolaemic saturated fatty acids (HSFA) was significantly higher in the organic system than in the other groups. Consequently, the h/H ratio, representing the ratio of hypocholesterolaemic to hypercholesterolaemic fatty acids, reached a significantly lower value in this system than in all others, which did not differ significantly from one another.

### 3.3. Other Bioactive Substances Analysis

The content of selected bioactive substances in eggs from different housing systems was also determined, as presented in Table 6. The analyses demonstrated a significant influence of the housing system on the content of all investigated compounds, with the exception of lysozyme, for which the differences were not fully consistent. Vitamin A content was significantly higher in the organic system compared to all other systems, which did not differ from one another. A similar pattern was observed for vitamin D, the highest level of which was again found in the organic system, which significantly exceeded the other groups, whereas the free-range, barn, and cage systems did not show significant differences between themselves. In the case of vitamin E, the organic system was characterised by a significantly higher content compared to all other systems, which did not differ from one another. Cholesterol content showed a different distribution among housing systems; significantly higher levels were recorded in the organic and free-range systems compared to the barn and cage systems. The hydrolytic activity of lysozyme was considerably higher in the organic system than in the barn and cage systems, whereas the free-range system did not differ significantly from any of the other groups.

The content of selected minerals in egg yolks was also determined, and the results are presented in Table 7. The analyses demonstrated a significant influence of the housing system on the content of all investigated elements, with the exception of arsenic, for which no significant differences between the systems were found. The most distinct trend was observed for magnesium, copper, and zinc, the content of which in the barn system was significantly higher than in the organic system; for magnesium, equally high levels were also found in the free-range and cage systems. In turn, calcium reached its highest values in the free-range and barn systems, significantly exceeding the organic system. In contrast, the highest potassium content was found in eggs from the organic group. The highest sodium content was recorded in egg yolks from the barn system, significantly exceeding the organic and cage systems in this respect. Regarding iron, the content in the barn system was significantly higher only in comparison to the cage system. In the case of lead, it is noteworthy that its content was significantly higher in organic eggs compared to the barn system, which may suggest greater exposure to this element under organic farming conditions. For cadmium, the differences between the systems proved to be statistically significant; however, due to the very low values detected (near the limit of detection), these differences are of formal rather than practical significance.

## 4. Discussion

The popularity of eggs from extensive systems continues to increase, driven by the prevailing consumer belief in their superior nutritional and health-promoting value. However, a comparison of the benefits and potential risks associated with various housing systems reveals that the reality is considerably more complex, and the choice of table eggs involves balancing multiple factors between bird welfare, product quality, and raw material safety.

Among all the analysed parameters, the exceptional albumen quality of eggs from the organic system, expressed in both Haugh units (92.03) and lysozyme activity (144,877 U/mL), merits particular attention. These values not only differed markedly from the results obtained for the cage system (70.86 and 119,830 U/mL, respectively) but also surpass the majority of literature reports. Philippe et al. [42] found no significant differences in Haugh units between housing systems (approx. 89–90), whereas Popova et al. [43] recorded a value of 75.1 for hens with outdoor access, which is comparable to the value obtained for the free-range system in the present study. This high lysozyme activity is particularly noteworthy given the enzyme’s well-documented role as a natural antimicrobial defence mechanism. Lysozyme acts by hydrolysing the β-1,4-glycosidic bonds between N-acetylmuramic acid and N-acetylglucosamine in the bacterial cell wall peptidoglycan layer, effectively providing a barrier against microbial spoilage. By maintaining such high enzymatic activity, eggs from organic systems may contribute to improved microbiological stability during storage [39,44]. Consequently, the superior quality traits observed in organic eggs, such as improved albumen height and integrity, are likely synergistic with their enhanced microbiological stability, thereby offering a multifaceted advantage in terms of food safety and shelf-life potential.

Most interestingly, however, Haugh units are directly correlated with thick albumen height, whose compact gel structure relies on the stability of the ovomucin–lysozyme complex. The stability of this complex is known to be highly dependent on pH, and the lower albumen pH in organic eggs (8.85 compared to 9.34 in the cage system) creates optimal conditions for maintaining its integrity. Furthermore, the higher lysozyme activity in this system, coupled with the appropriate pH, may effectively support the structural stability of the albumen gel. These enhanced quality traits are likely reflections of the overall physiological and immunological status of hens maintained under extensive management conditions [38].

The mechanism underlying this phenomenon is most likely twofold. On the one hand, hens in the organic system, having free access to outdoor runs, exhibit higher physical activity, which influences calcium metabolism and overall physiological condition, and indirectly, albumen quality [16,17]. On the other hand, increasing attention is being paid to the role of the gut microbiome in shaping the quality of animal-derived products. As indicated by Pires et al. [45] in a comprehensive systematic review, alternative systems are characterised by greater alpha diversity of the gut microbiome compared to cage systems. Furthermore, studies by Wan et al. [46] and Gan et al. [47] have demonstrated that certain gut bacteria are positively correlated with Haugh units. Hens in the organic system, by developing a richer microbiome through access to soil, plants, and the natural environment, may produce eggs with higher albumen quality. Consequently, the higher lysozyme activity in organic eggs may be not only the result of direct immunological stimulation arising from greater pathogen pressure in the outdoor environment [22], but also an indirect effect of microbiome modulation supporting the hen’s overall immunological condition. Although this hypothesis is based on recent literature reports and remains speculative, it is consistent with the superior albumen quality characteristics observed in the present study. It should be noted that no microbiome analysis was performed in this trial. As Pires et al. [45] emphasise that research on the microbiome within the organic system is currently completely absent, further studies are needed to empirically verify the potential relationship between the housing system, gut microbiota, and egg albumen quality. Although direct microbiological parameters were not evaluated in the present study, it can be hypothesised that variations in feeding behaviour and environmental exposure across housing systems might theoretically influence digestive processes and nutrient utilisation. While the specific contributions of bacterial taxa (such as *Phascolarctobacterium* or *Bacteroides* [39] or *Lactobacillus* [48]) to other parameters like shell matrix formation or lipid metabolism remain to be elucidated in targeted future research, such physiological pathways offer a valuable theoretical background for understanding the observed phenotypic differences.

Paradoxically, however, the highest albumen quality was accompanied by the lowest shell breaking strength (34.19 N), a value considerably lower than in the free-range (47.82 N) and cage (47.05 N) systems, but comparable to the results of Minelli et al. [19] for organic eggs. Shell thickness in the organic system (0.299 mm) proved to be significantly lower than in the cage system (0.361 mm), consistent with the observations by Yenice et al. [18].

This apparent contradiction, high albumen quality coupled with a weaker shell in the organic system, is likely multifactorial. On one hand, some authors hypothesise that greater physical activity in extensive systems stimulates bone remodelling, potentially causing calcium to be preferentially allocated to bone conservation at the expense of the eggshell [49]. On the other hand, a highly plausible mechanism in outdoor-access systems is dietary calcium dilution [50]. In laying hens, daily shell formation relies heavily on medullary bone turnover and adequate dietary calcium intake. The substantial consumption of moisture-rich, low-calcium outdoor forage and insects by organic hens likely dilutes their overall daily calcium intake relative to their production output, thereby compromising shell strength.

Additionally, as indicated by the research of Lichovniková and Zeman [50], calcium intake in barn systems is lower than in cage systems. This is corroborated by the lowest Ca content found in organic yolks in the present study compared to the cage system (2081 mg/kg versus 2636 mg/kg). A particularly valuable comparison emerges between the barn and free-range systems, where the hens were fed an identical commercial diet. Despite sharing the exact same dietary calcium and phosphorus baseline, free-range eggs exhibited significantly higher shell breaking strength and thickness than barn eggs. This directly highlights the beneficial impact of the outdoor run; exposure to natural sunlight enhances endogenous vitamin D synthesis, thereby improving intestinal calcium absorption and shell matrix mineralization compared to strict indoor confinement [4].

The yolk and shell colour also align with the presented pattern of relationships. The darker yolk colour in the barn and free-range systems compared to the organic system may appear surprising in light of the consumer perception that eggs from outdoor-access systems possess the most intense colour. However, these differences can likely be attributed to the use of synthetic colouring additives, such as xanthophylls, in commercial feeds (particularly in the barn and cage systems), the use of which is restricted in organic farming [21,51]. Furthermore, comparing the barn and free-range systems, which shared an identical basal diet, reveals a direct “outdoor effect.” Despite ingesting the same synthetic pigments from the identical feed, free-range yolks were significantly lighter. This pigment dilution in the free-range system is most likely driven by their significantly higher yolk weight, coupled with the supplementary consumption of unpigmented natural matter (e.g., soil and insects) from the outdoor run. In turn, the darkest shell colour in the cage system (22.73% of light reflectance) most likely results from the hens’ genotype, rather than from feed additives or the housing system itself.

An equally complex picture emerges from the analysis of the fatty acid profile. Eggs from the organic system contained significantly higher levels of α-linolenic acid (ALA) and docosahexaenoic acid (DHA), n-3 family compounds with a documented beneficial impact on the cardiovascular system and cognitive functions [2]. The DHA content in organic eggs (0.673%) was almost twice as high as in the free-range (0.340%) and cage (0.360%) systems, which is in agreement with the results of Mierliță [52] and Popova et al. [43]. The explanation for this phenomenon is relatively straightforward: the green forage on outdoor runs contains up to 21% ALA in dry matter [52], which is a precursor to DHA, and organic hens have unrestricted access to it. Furthermore, birds on the run also consume insects rich in long-chain n-3 fatty acids, which could further contribute to the enrichment of yolk lipids with these compounds [53].

Simultaneously, however, organic eggs were characterised by the highest saturated fatty acid (SFA) content, including palmitic (24.12%) and stearic (7.84%) acids. This apparent contradiction, higher levels of both beneficial n-3 and less favourable SFAs, results from two mechanisms. Firstly, hens on outdoor runs consume more total fat (both saturated and unsaturated) from green forage and insects, which translates into a higher total lipid content in the yolk. Secondly, although some literature suggests that outdoor systems can exhibit varying lipid pool dynamics depending on flock characteristics [54], in the present study the cage system actually showed a slightly higher yolk proportion (27.10%) than the organic system (24.24%). Therefore, the elevated SFA content in organic eggs is not related to morphological component proportions, but is primarily driven by specific dietary inputs, the lipid profile of ingested natural forage and insects on the outdoor run, and the distinct metabolic utilisation characteristic of organic farming.

Consequently, the lipid profile quality indices, such as the Atherogenicity Index (AI–0.411), Thrombogenicity Index (TI–3.455), and the ratio of hypocholesterolaemic to hypercholesterolaemic fatty acids (h/H–2.404), were less favourable in the organic system than in the remaining systems. As emphasised by Ulbricht and Southgate [32], these dimensionless parameters are theoretical compositional indices reflecting the inherent balance of the fat fraction and its potential dietary propensity to influence cardiovascular risk, rather than direct measures of clinical outcomes or absolute intake per serving. This implies that while organic egg fat possesses a unique proportional fatty acid distribution, these indices serve as compositional indicators of lipid quality rather than direct clinical predictors. These values are comparable to the results reported by Mugnai et al. [53] and Popova et al. [43]. This implies that, although a consumer selecting an organic egg gains more DHA and ALA, the overall lipid profile, assessed through the lens of consolidated indices, is simultaneously less favourable than that of eggs from cage or barn systems. This apparent contradiction is crucial for understanding the limitations of simplistic marketing messages: an organic egg is not universally healthier than a cage-system egg in every aspect. It is simply different, richer in certain components but poorer in others, and the ultimate balance depends on which nutritional aspect is deemed a priority.

Analogous mechanisms underlie the differences in vitamin content. Eggs from the organic system contained significantly more vitamins A, D, and E compared to all other systems. Vitamin A reached a level of 1600 μg/100 g, a value almost twice as high as in the remaining systems and exceeding the data reported by Anderson [55]. Similarly, vitamin E (3267 μg/100 g) was significantly higher than in the cage system (2300 μg/100 g), which is corroborated by Mierliță [52]. The mechanism behind this advantage is multifactorial and partially overlaps with the mechanisms responsible for the higher n-3 fatty acid content. Access to outdoor green forage provides hens with natural sources of carotenoids (provitamin A) and tocopherols (vitamin E), which are subsequently deposited in the yolk [36], while exposure to sunlight stimulates the endogenous synthesis of vitamin D [4]. Crucially, green forage is rich in fibre and polyphenols, which, as recent studies suggest, can modulate the composition of the gut microbiome, increasing the efficiency of nutrient extraction from feed and supporting the absorption of fat-soluble vitamins [56]. In the cage system, despite full dietary control, the feed lacks natural green components rich in bioactive compounds; even if it contains vitamin supplements, the hen’s ability to absorb and deposit them may be limited by a less efficient microbiome [57]. The higher cholesterol content in eggs from outdoor systems (10.52–11.32 mg/g) than in indoor systems (8.21–8.57 mg/kg) is primarily driven by the higher proportion of yolk relative to the total egg mass.

However, the most concerning result of the present study is the significantly higher lead content in organic eggs (0.361 mg/kg) compared with eggs from the barn (0.041 mg/kg) and cage (0.102 mg/kg) systems. This result is consistent with our previous findings [24], which demonstrated that organic eggs can serve as bioindicators of environmental heavy metal contamination. Similar observations were presented by Radu-Rusu et al. [58] and Tanhan et al. [59]. Crucially, the lead level in organic eggs was higher than in the study by Demirulus [60] for rural areas (approx. 0.05–0.10 mg/kg), indicating local contamination, likely particulate deposition from nearby emission sources. Hens in the organic system, having free access to outdoor runs, ingest feed (including green forage, insects, and soil) from surfaces where environmental contaminants may have been deposited. As demonstrated in the correlation analysis, the Pb content in organic eggs was significantly negatively correlated with Ca, Mg, and Zn, suggesting that lead competes with these elements for binding sites within the hen’s body [61]. This mechanism is of crucial importance for consumer safety: a higher Pb level in organic eggs implies that a nutritionally superior egg may simultaneously be more contaminated. Importantly, differences in the gut microbiome between systems may also affect the bioavailability and toxicity of heavy metals; certain gut bacteria can bind metals, thereby limiting their absorption, whereas others may increase their solubility and toxicity [45]. This represents another area where microbiological mechanisms may mediate the transfer of contaminants into the egg. The cage system, by eliminating contact with soil, appears to offer the highest level of chemical safety, though at the expense of albumen quality and vitamin content.

It is crucial to highlight that, currently, there are no established legal safety standards (Maximum Permissible Levels) for heavy metals in chicken eggs within the European Union [62]. The absence of such regulatory limits represents a significant gap in food safety legislation. Given that our results, along with previous studies, indicate that alternative farming systems are particularly prone to the accumulation of lead from the environment, the lack of specific standards may lull consumers and regulatory bodies into a false sense of security. Therefore, it is highly advisable to advocate for regular toxicological monitoring of eggs originating from free-range and organic systems.

## 5. Conclusions

The laying hen housing system is strongly associated with the multidimensional quality of table eggs, simultaneously influencing physical traits, the bioactive profile, and chemical safety. These results confirm that egg assessment should be based on an integrated approach, extending beyond the analysis of single quality parameters. However, because these findings derive from a single geographical region, a limited number of commercial suppliers, and a single sampling period, they should not be generalised as universal rules for all production systems.

Eggs from the organic system stand out for their highest albumen quality, as corroborated by physical parameters (high Haugh units) and high lysozyme activity. This result indicates the potential of this system for naturally extending the shelf life of the raw material and increasing its resistance to structural degradation.

The organic housing system promotes the deposition of valuable substances, such as n-3 polyunsaturated fatty acids (including DHA) and fat-soluble vitamins (A, D, E). Simultaneously, this system modifies the lipid profile in a manner that results in different values for the atherogenicity (AI) and thrombogenicity (TI) indices compared to the cage and barn systems. This highlights that "higher nutritional value" is a complex category, requiring consumers to consciously balance their dietary priorities.

The higher lead content in organic egg yolks raises important food safety concerns, confirming that under extensive farming conditions (free-range and organic), eggs can serve as bioindicators of environmental contamination. This result indicates the necessity for stricter supervision of the outdoor run environment’s quality and the implementation of better chemical risk management strategies in alternative systems.

In conclusion, rather than supporting broad assertions that organic housing universally enhances nutritional value or that cage housing inherently guarantees chemical safety, our findings show that the choice of a housing system entails the necessity of accepting a complex trade-off between health-promoting benefits and potential regional risks. Our research highlights the need for consumer education that moves beyond simplistic marketing messages in favour of transparent knowledge about quality and safety, thereby supporting informed purchasing decisions.

## Figures and Tables

**Table 1:** The proportions of particular egg and/or eggshell defects found in eggs derived from various rearing systems of hens (%) *.

Defect	The Rearing System					χ^2^ (*p*-Value)
	Organic (0)	Free-Range (1)	Barn (2)	Cage (3)	Total	
intact	9.04	4.55	0.56	2.88	4.31	0.003
external cracks	1.81	3.64	0.56	2.88	1.97	0.295
internal weak cracks	0.00	3.64	2.26	2.88	1.97	0.154
internal strong cracks	1.81	7.27	1.13	5.77	3.41	0.019
severe stripe marks	16.87	19.09	7.34	23.08	15.44	0.011
points	27.11	38.18	2.82	43.27	24.60	0.000
wrinkled	0.00	4.55	10.17	2.88	4.67	0.000
marbled	7.23	0.00	8.47	3.85	5.57	0.019
movable air cell	1.20	0.00	0.00	0.00	0.36	0.197
weak ends	5.42	2.73	15.82	3.85	7.90	0.000
broken chalaza	21.08	11.82	15.82	5.77	14.72	0.020
abnormal shape	6.02	0.00	10.17	0.96	5.21	0.001
pimples	2.41	4.55	24.86	1.92	9.87	0.000

* Statistical analysis (chi-squared test) was performed on absolute counts (number of eggs with a given defect).

**Table 2:** Whole egg quality traits depending on the hens’ rearing system.

Trait		The Rearing System								F Test (*p*-Value)
		Organic (0)		Free-Range (1)		Barn (2)		Cage (3)		
		$\bar{x}$	SD	$\bar{x}$	SD	$\bar{x}$	SD	$\bar{x}$	SD	
Weight (g)		50.83 ^a^	7.921	64.08 ^d^	2.456	60.37 ^c^	2.054	57.69 ^b^	1.891	<0.001
Shape index		0.75 ^b^	0.038	0.77 ^b^	0.022	0.74 ^a^	0.028	0.78 ^c^	0.028	0.033
Specific gravity (g/cm^3^)		1.080	0.008	1.086	0.006	1.117	0.277	1.085	0.007	0.050
Proportion (%)	yolk	24.24 ^a^	3.892	26.98 ^a^	1.943	26.80 ^a^	2.101	27.10 ^b^	1.813	0.006
	albumen	62.60 ^c^	5.348	60.26 ^b^	1.847	60.54 ^b^	2.510	59.87 ^a^	1.773	0.016
	shell	13.17	2.653	12.74	0.579	12.73	1.187	13.03	0.817	0.083

^a,b,c,d^—mean differ significantly at *p* ≤ 0.05; SD—standard deviation.

**Table 3:** The eggshell quality traits depending on the hens’ rearing system.

Trait	The Rearing System								F Test (*p*-Value)
	Organic (0)		Free-Range (1)		Barn (2)		Cage (3)		
	$\bar{x}$	SD	$\bar{x}$	SD	$\bar{x}$	SD	$\bar{x}$	SD	
breaking strength (N)	34.19 ^a^	8.502	47.82 ^b^	12.460	34.40 ^a^	9.570	47.05 ^b^	14.608	0.035
colour (%)	48.87 ^b^	10.749	51.22 ^b^	4.687	59.50 ^c^	10.169	22.73 ^a^	4.087	<0.001
weight (g)	6.72 ^a^	1.827	8.16 ^c^	0.446	7.68 ^bc^	0.737	7.51 ^b^	0.500	<0.001
thickness (mm)	0.299 ^a^	0.040	0.338 ^b^	0.026	0.299 ^a^	0.027	0.361 ^c^	0.057	0.034
area (cm^2^)	64.97 ^a^	6.698	75.93 ^d^	1.918	72.99 ^c^	1.652	70.82 ^b^	1.544	<0.001
volume (cm^3^)	1.95 ^ac^	0.370	2.56	0.222	2.18 ^b^	0.195	2.55 ^c^	0.407	0.038
density (g/cm^3^)	3.46	0.763	3.20	0.263	3.54	0.375	3.15	1.637	0.083

^a,b,c,d^—means differ significantly at *p* ≤ 0.05; SD—standard deviation.

**Table 4:** The egg content quality traits depending on the hens’ rearing system.

Trait	The Rearing System								F Test (*p*-Value)
	Organic (0)		Free-Range (1)		Barn (2)		Cage (3)		
	$\bar{x}$	SD	$\bar{x}$	SD	$\bar{x}$	SD	$\bar{x}$	SD	
**Albumen**									
weight (g)	31.80 ^a^	5.923	38.61 ^d^	2.170	36.56 ^c^	2.096	34.52 ^b^	1.634	<0.001
height (mm)	8.16 ^d^	1.672	6.13 ^c^	1.131	4.98 ^a^	1.536	5.31 ^b^	1.555	0.002
Haugh units	92.03 ^d^	8.534	75.50 ^c^	7.464 ^a^	66.34 ^a^	11.973	70.86 ^b^	9.676	0.003
pH	8.85 ^ab^	0.240	8.76 ^a^	0.090	9.02 ^ab^	0.178	9.34 ^b^	0.081	0.039
**Yolk**									
weight (g)	12.29 ^a^	2.796	17.26 ^c^	1.243	16.18 ^b^	1.328	15.62 ^b^	1.148	<0.001
index	0.419 ^b^	0.042	0.402 ^b^	0.063	0.403 ^b^	0.058	0.375 ^a^	0.039	0.034
colour (pts)	7.55 ^a^	1.819	10.88 ^b^	0.904	11.95 ^c^	1.395	10.55 ^b^	1.172	0.019
pH	6.20 ^a^	0.143	5.85 ^a^	0.519	5.99 ^b^	0.111	6.11 ^c^	0.107	0.030
**Others**									**χ^2^** **(*p*-Value)**
meat spots (%)	23.28		32.58		21.05		34.78		0.001
blood spots (%)	12.75		24.05		15.49		7.69		0.001

^a,b,c,d^—means differ significantly at *p* ≤ 0.05; SD—standard deviation.

**Table 5:** The indexes of fatty acids in yolk depending on the hens’ rearing system.

Trait	The Rearing System								F Test (*p*-Value)
	Organic (0)		Free-Range (1)		Barn (2)		Cage (3)		
	$\bar{x}$	SD	$\bar{x}$	SD	$\bar{x}$	SD	$\bar{x}$	SD	
SFA	32.62 ^b^	1.738	29.37 ^a^	0.766	30.35 ^a^	1.375	29.84 ^a^	0.722	<0.001
MUFA	45.27 ^a^	1.759	45.28 ^a^	2.067	46.43 ^ab^	3.326	48.16 ^b^	2.374	0.018
PUFA	17.36 ^a^	2.179	20.83 ^b^	2.781	18.87 ^ab^	3.715	17.29 ^a^	2.685	0.014
n-3	1.488 ^b^	0.442	0.552 ^a^	0.188	0.493 ^a^	0.251	0.390 ^a^	0.184	<0.001
n-6	15.87 ^a^	1.857	20.28 ^b^	2.619	18.38 ^a^	3.485	16.90 ^a^	2.574	0.002
n-9	41.69 ^a^	1.754	42.77 ^a^	1.819	43.86 ^ab^	3.476	45.56 ^b^	2.194	0.002
PI	27.18	3.890	27.27	3.717	25.45	4.915	23.23	3.764	0.670
AI	0.411 ^b^	0.026	0.350 ^a^	0.012	0.366 ^a^	0.024	0.357 ^a^	0.018	<0.001
TI	3.455 ^b^	0.494	2.765 ^a^	0.402	3.251 ^ab^	0.827	3.427 ^b^	0.612	0.028
DFA	70.47 ^a^	1.027	72.98 ^b^	0.691	72.45 ^b^	0.957	72.61 ^b^	0.980	<0.001
HSFA	24.51 ^b^	0.982	22.24 ^a^	0.483	22.97 ^a^	1.149	22.45 ^a^	0.868	<0.001
h/H	2.404 ^a^	0.160	2.847 ^b^	0.102	2.727 ^b^	0.188	2.792 ^b^	0.150	<0.001

SFA—saturated fatty acids, MUFA—monounsaturated fatty acids, PUFA—polyunsaturated fatty acids, PI—peroxidizability index, AI—atherogenicity index, TI—thrombogenic index, DFA—desirable fatty acids, HSFA—hypercholesterolaemic saturated fatty acids, h/H—hypocholesterolaemic/hypercholesterolaemic ratio; ^a,b^—means in the row differ significantly at *p* ≤ 0.05; SD—standard deviation.

**Table 6:** Bioactive substances in the egg content depending on the hens’ rearing system.

Trait	The Rearing System								F Test (*p*-Value)
	Organic (0)		Free-Range (1)		Barn (2)		Cage (3)		
	$\bar{x}$	SD	$\bar{x}$	SD	$\bar{x}$	SD	$\bar{x}$	SD	
Vitamin A (μg/100 g)	1600 ^b^	829	954 ^a^	304	812 ^a^	502	991 ^a^	451	0.006
Vitamin D (μg/100 g)	17.32 ^b^	2.21	13.47 ^a^	3.36	11.83 ^a^	1.50	12.52 ^a^	1.18	0.000
Vitamin E (μg/100 g)	3267 ^b^	1394	2250 ^a^	720	1975 ^a^	795	2300 ^a^	1090	0.021
Cholesterol (mg/g)	10.52 ^b^	1.57	11.32 ^b^	1.01	8.57 ^a^	2.74	8.21 ^a^	1.27	0.000
Lysozyme (U/mL)	144,877 ^b^	46,838	127,330 ^ab^	44,561	121,564 ^a^	48,813	119,830 ^a^	52,031	0.002

^a,b^—means differ significantly at *p* ≤ 0.05; SD—standard deviation.

**Table 7:** Mineral composition of egg yolks [mg/kg] depending on the hens’ rearing system.

Trait	The Rearing System								F Test (*p*-Value)
	Organic (0)		Free-Range (1)		Barn (2)		Cage (3)		
	$\bar{x}$	SD	$\bar{x}$	SD	$\bar{x}$	SD	$\bar{x}$	SD	
Ca	2081.3 ^a^	173.7	2673.3 ^b^	221.5	3021.7 ^b^	287.8	2636.3 ^b^	843.0	<0.001
K	2451.3 ^b^	349.9	2030.0 ^a^	68.4	2116.7 ^ab^	183.2	1972.2 ^a^	532.5	<0.001
Mg	225.0 ^a^	40.8	303.0 ^b^	28.6	297.7 ^b^	47.1	280.5 ^b^	81.2	<0.001
Na	809.4 ^a^	68.7	911.7 ^ab^	94.3	961.0 ^b^	170.5	789.7 ^a^	231.8	0.011
Fe	110.8 ^ab^	7.6	109.0 ^ab^	12.1	113.8 ^b^	9.00	95.5 ^a^	27.5	0.019
Cu	2.953 ^a^	0.433	2.773 ^a^	0.254	3.762 ^b^	1.360	2.400 ^a^	0.699	<0.001
Zn	54.66 ^a^	5.18	64.63 ^a^	3.99	84.58 ^b^	31.07	60.98 ^a^	18.49	<0.001
Pb	0.361 ^b^	0.445	0.118 ^ab^	0.082	0.041 ^a^	0.024	0.102 ^ab^	0.103	0.006
Cd	0.001	0.001	0.000	0.000	0.002	0.003	0.000	0.000	0.011
As	0.000	0.000	0.002	0.005	0.049	0.098	0.035	0.085	0.108

Ca—calcium, K—potassium, Mg—magnesium, Na—sodium, Fe—iron, Cu—copper, Zn—zinc, Pb—lead, Cd—cadmium, As—arsenic; ^a,b^—means differ significantly at *p* ≤ 0.05; SD—standard deviation.

## Data Availability

Data presented in this study are available from the corresponding author on the request.

## Supplementary Materials

The following supporting information can be downloaded at: https://www.mdpi.com/article/10.3390/foods15162835/s1, Table S1: The basic composition (%) of feed mixtures depending on bird’s rearing system; Table S2: The fatty acids content in the samples feed mixtures (%); Table S3: The heavy metals content in the samples feed mixtures; Table S4: The proportion of particular fatty acids in egg yolks depending on bird’s rearing system.

## Abbreviations

The following abbreviations are used in this manuscript:

AI	atherogenicity index
As	arsenic
Ca	calcium
Cd	cadmium
Cu	copper
DFA	desirable fatty acids
Fe	iron
HSFA	hypercholesterolaemic saturated fatty acids
h/H	hypocholesterolaemic/hypercholesterolaemic ratio
K	potassium
LOD	Limit of detection
LOQ	Limit of quantification
Mg	magnesium
MUFA	monounsaturated fatty acids
Na	sodium
Pb	lead
PI	peroxidizability index
PUFA	polyunsaturated fatty acids
SD	standard deviation
SFA	saturated fatty acids
TI	thrombogenic index
Zn	zinc

## References

[1] FAO (2023). Contribution of Terrestrial Animal Source Food to Healthy Diets for Improved Nutrition and Health Outcomes.

[2] Shinn S.E., Proctor A., Baum J.I. (2018). Egg Yolk as Means for Providing Essential and Beneficial Fatty Acids. J. Am. Oil Chem. Soc..

[3] Pal M., Molnár J. (2021). The Role of Eggs as an Important Source of Nutrition in Human Health. IJFSA.

[4] Clark A., Kuznesof S., Davies S., Waller A., Ritchie A., Wilson S., Harbord L., Hill T. (2021). Egg Enrichment with Vitamin D: The Sunshine Eggs Projects. Nutr. Bull..

[5] Białowąs W., Blicharska E., Drabik K. (2024). Biofortification of Plant- and Animal-Based Foods in Limiting the Problem of Microelement Deficiencies—A Narrative Review. Nutrients.

[6] (1999). Council Directive 1999/74/EC of 19 July 1999 laying down minimum standards for the protection of laying hens. Off. J. Eur. Communities.

[7] European Union (2002). Commission Directive 2002/4/EC of 30 January 2002 on the registration of establishments keeping laying hens, covered by Council Directive 1999/74/EC. Off. J. Eur. Communities.

[8] (2023). Commission Implementing Regulation (EU) 2023/2466 of 17 August 2023 laying down rules for the application of Regulation (EU) No 1308/2013 of the European Parliament and of the Council as regards marketing standards for eggs. Off. J. Eur. Union.

[9] Drabik K., Spasowska-Czarny H., Batkowska J. (2024). The Freshness and Quality Indicators of Table Eggs During Their Storage on the Background of Existing Legislation. Prz. Prawa Adm..

[10] Eurostat Production of Eggs for Consumption and Number of Laying Hens (2013–2024). https://ec.europa.eu/eurostat/databrowser/view/APRO_EC_EGGHEN/default/table?lang=en.

[11] Pires P.G.D.S., Bavaresco C., Prato B.S., Wirth M.L., Moraes P.D.O. (2021). The Relationship between Egg Quality and Hen Housing Systems—A Systematic Review. Livest. Sci..

[12] Windhorst H.W. (2017). Dynamics and patterns of the EU egg industry. Lohmann Inf..

[13] Nielsen S.S., Alvarez J., Bicout D.J., Calistri P., Canali E., Drewe J.A., Garin-Bastuji B., Gonzales Rojas J.L., Gortázar Schmidt C., EFSA Panel on Animal Health and Animal Welfare (AHAW) (2023). Welfare of Laying Hens on Farm. EFS2.

[14] European Commission European Citizens’ Initiative: End the Cage Age. https://food.ec.europa.eu/animals/animal-welfare/eci/eci-end-cage-age_en.

[15] Batkowska J., Drabik K., Brodacki A. (2017). Quantity and Quality of Poultry Products Depending on Birds’ Rearing System. J. Anim. Sci. Biol. Bioecon..

[16] Tůmová E., Ebeid T. (2005). Effect of Time of Oviposition on Egg Quality Characteristics in Cages and in a Litter Housing System. Czech. J. Anim. Sci..

[17] Van Den Brand H., Parmentier H.K., Kemp B. (2004). Effects of Housing System (Outdoor *vs* Cages) and Age of Laying Hens on Egg Characteristics. Brit. Poult. Sci..

[18] Yenice G., Kaynar O., Ileriturk M., Hira F., Hayirli A. (2016). Quality of Eggs in Different Production Systems. Czech. J. Food Sci..

[19] Minelli G., Sirri F., Folegatti E., Meluzzi A., Franchini A. (2007). Egg Quality Traits of Laying Hens Reared in Organic and Conventional Systems. Ital. J. Anim. Sci..

[20] Rakonjac S., Dosković V., Bošković S., Škrbić Z., Lukić M., Petričević V., Petrović D. (2021). Production Performance and Egg Quality of Laying Hens as Influenced by Genotype and Rearing System. Braz. J. Poult. Sci..

[21] Dvořák P., Suchý P., Straková E., Doležalová J. (2010). Variation in Egg Yolk Colour in Different Systems of Rearing Laying Hens. Acta Vet. Brno.

[22] Parisi M.A., Northcutt J.K., Smith D.P., Steinberg E.L., Dawson P.L. (2015). Microbiological Contamination of Shell Eggs Produced in Conventional and Free-Range Housing Systems. Food Control.

[23] Gondek M., Szkucik K., Bełkot Z. (2013). Wpływ Różnych Systemów Utrzymania Kur Na Zanieczyszczenie Powierzchni Jaj Bakteriami Chorobotwórczymi. Med. Weter..

[24] Drabik K., Skałecki P., Spasowska H., Melnyk O.P., Kutrzuba M., Batkowska J. (2024). Organic Eggs as an Indirect Indicator of Agricultural Environmental Pollution—Preliminary Research. J. Food Comp. Anal..

[25] Li X., Xia M., Zeng Q., Zhang X., Sun H., Huang X., Ahn D.U., Salama M., Mourad F.K., Cai Z. (2023). Eggstraordinary Health: Exploring Avian Egg Proteins and Peptides in Boosting Immunity and Health Maintenance. Food Sci. Anim. Prod..

[26] Li X., Li Z., Salama M., Huang X., Wang Y., Cai Z. (2024). Eggceptional Variances: Exploring Immunomodulation across Commercial Caged, Free-Range, and Tibetan Environments. Food Biosci..

[27] Wengerska K., Batkowska J., Drabik K. (2023). The Eggshell Defect as a Factor Affecting the Egg Quality after Storage. Poult. Sci..

[28] Shafey T.M. (2002). Effects of Egg Size and Eggshell Conductance on Hatchability Traits of Meat and Layer Breeder Flocks. Asian-Australas. J. Anim. Sci..

[29] Williams K.C. (1992). Some Factors Affecting Albumen Quality with Particular Reference to Haugh Unit Score. World Poult. Sci. J..

[30] Batkowska J., Drabik K., Brodacki A., Czech A., Adamczuk A. (2021). Fatty Acids Profile, Cholesterol Level and Quality of Table Eggs from Hens Fed with the Addition of Linseed and Soybean Oil. Food Chem..

[31] Arakawa K., Sagai M. (1986). Species Differences in Lipid Peroxide Levels in Lung Tissue and Investigation of Their Determining Factors. Lipids.

[32] Ulbricht T.L.V., Southgate D.A.T. (1991). Coronary Heart Disease: Seven Dietary Factors. Lancet.

[33] Medeiros E., Queiroga R., Oliveira M., Medeiros A., Sabedot M., Bomfim M., Madruga M. (2014). Fatty Acid Profile of Cheese from Dairy Goats Fed a Diet Enriched with Castor, Sesame and Faveleira Vegetable Oils. Molecules.

[34] Renna M., Cornale P., Lussiana C., Malfatto V., Mimosi A., Battaglini L.M. (2012). Fatty Acid Profile of Milk from Goats Fed Diets with Different Levels of Conserved and Fresh Forages. Int. J. Dairy Tech..

[35] Domaradzki P., Florek M., Skałecki P., Litwińczuk A., Kędzierska-Matysek M., Wolanciuk A., Tajchman K. (2019). Fatty Acid Composition, Cholesterol Content and Lipid Oxidation Indices of Intramuscular Fat from Skeletal Muscles of Beaver (*Castor fiber* L.). Meat Sci..

[36] Mattila P., Lehikoinen K., Kiiskinen T., Piironen V. (1999). Cholecalciferol and 25-Hydroxycholecalciferol Content of Chicken Egg Yolk As Affected by the Cholecalciferol Content of Feed. J. Agric. Food Chem..

[37] Kasperek K., Drabik K., Zięba G., Batkowska J. (2023). The Quality of Eggs Derived from Polbar and Greenleg Partridge Hens—Polish Conservative Breeds. Acta Sci. Pol. Zootech..

[38] Drabik K., Próchniak T., Kasperek K., Batkowska J. (2021). The Use of the Dynamics of Changes in Table Eggs during Storage to Predict the Age of Eggs Based on Selected Quality Traits. Animals.

[39] Wengerska K., Spustek D., Krakowiak D., Drabik K., Batkowska J. (2021). Rearing System, Utility Type and Age of Hens as Factors Modifying the Hydrolytic Activity of Lysozyme. Rocz. Nauk. Pol. Tow. Zootech..

[40] AOAC (2019). Official Methods of Analysis of AOAC International.

[41] IBM SPSS Inc. (2017). SPSS Statistics for Windows, Version 24.0.

[42] Philippe F.X., Mahmoudi Y., Cinq-Mars D., Lefrançois M., Moula N., Palacios J., Pelletier F., Godbout S. (2020). Comparison of Egg Production, Quality and Composition in Three Production Systems for Laying Hens. Livest. Sci..

[43] Popova T., Petkov E., Ayasan T., Ignatova M. (2020). Quality of Eggs from Layers Reared under Alternative and Conventional System. Braz. J. Poult. Sci..

[44] Banaszewska D., Biesiada-Drzazga B., Ostrowski D., Drabik K., Batkowska J. (2019). The Impact of Breeder Age on Egg Quality and Lysozyme Activity. Turk. J. Vet. Anim. Sci..

[45] Pires P.G.S., Oliveira G.S., McManus C., Santos V.M., Moraes P.O. (2024). Impact of Housing System on Intestinal Microbiota of Laying Hens—A Systematic Review. Res. Vet. Sci..

[46] Wan Y., Ma R., Zhang H., Li L., Chai L., Qi R., Liu W., Li J., Li Y., Zhan K. (2021). Different Non-Cage Housing Systems Alter Duodenal and Cecal Microbiota Composition in Shendan Chickens. Front. Vet. Sci..

[47] Gan L., Zhao Y., Mahmood T., Guo Y. (2020). Effects of Dietary Vitamins Supplementation Level on the Production Performance and Intestinal Microbiota of Aged Laying Hens. Poult. Sci..

[48] Choe D.W., Loh T.C., Foo H.L., Hair-Bejo M., Awis Q.S. (2012). Egg Production, Faecal pH and Microbial Population, Small Intestine Morphology, and Plasma and Yolk Cholesterol in Laying Hens given Liquid Metabolites Produced by *Lactobacillus plantarum* Strains. Br. Poult. Sci..

[49] Scholz B., Rönchen S., Hamann H., Sürie C., Neumann U., Kamphues J., Distl O. (2008). Evaluation of Bone Strength, Keel Bone Deformity and Egg Quality of Laying Hens Housed in Small Group Housing Systems and Furnished Cages in Comparison to an Aviary Housing System. Arch. Anim. Breed..

[50] Lichovníková M., Zeman L. (2008). Effect of Housing System on the Calcium Requirement of Laying Hens and on Eggshell Quality. Czech. J. Anim. Sci..

[51] Sokołowicz Z., Krawczyk J., Dykiel M. (2018). Effect of Alternative Housing System and Hen Genotype on Egg Quality Characteristics. Emir. J. Food Agric..

[52] Mierliță D. (2020). Fatty Acid Profile and Oxidative Stability of Egg Yolks from Hens under Different Production Systems. S. Afr. J. Anim. Sci..

[53] Mugnai C., Sossidou E.N., Dal Bosco A., Ruggeri S., Mattioli S., Castellini C. (2014). The Effects of Husbandry System on the Grass Intake and Egg Nutritive Characteristics of Laying Hens. J. Sci. Food Agric..

[54] Kralik Z., Radišić Z., Grčević M., Kralik G. Comparison of table eggs quality originating from hens kept in different housing systems. Proceedings of the XXI European Symposium on the Quality of Poultry Meat and XV European Symposium on the Quality of Eggs and Egg Products.

[55] Anderson K.E. (2011). Comparison of Fatty Acid, Cholesterol, and Vitamin A and E Composition in Eggs from Hens Housed in Conventional Cage and Range Production Facilities. Poult. Sci..

[56] Kang R., Wang W., Liu Y., Huang S., Xu J., Zhao L., Zhang J., Ji C., Wang Z., Hu Y. (2022). Dietary Selenium Sources Alleviate Immune Challenge Induced by Salmonella Enteritidis Potentially through Improving the Host Immune Response and Gut Microbiota in Laying Hens. Front. Immunol..

[57] Küçükyılmaz K., Bozkurt M., Herken E.N., Çınar M., Çatlı A.U., Bintaş E., Çöven F. (2012). Effects of Rearing Systems on Performance, Egg Characteristics and Immune Response in Two Layer Hen Genotype. Asian-Australas. J. Anim. Sci..

[58] Radu-Rusu C.G., Pop I.M., Albu A., Bologa M., Radu-Rusu R.M. (2013). Transferability of Certain Heavy Metals from Hens Feed to Table Eggs Laid within Different Rearing Systems. Lucr. Ştiinţ. Ser. Zooteh..

[59] Tanhan P., Apipongrattanasuk N., Poapolathep A., Poapolathep S., Kruatrachue M., Imsilp K. (2020). Heavy metal concentrations in duck eggs and potential human health risk via consumption. Jpn. J. Vet. Res..

[60] Demirulus H. (2013). The Heavy Metal Content in Chicken Eggs Consumed in Van Lake Territory. Ekoloji.

[61] Qin S., Liu H., Nie Z., Rengel Z., Gao W., Li C., Zhao P. (2020). Toxicity of Cadmium and Its Competition with Mineral Nutrients for Uptake by Plants: A Review. Pedosphere.

[62] (2023). Commission Regulation (EU) 2023/915 of 25 April 2023 on maximum levels for certain contaminants in food and repealing Regulation (EC) No 1881/2006. Off. J. Eur..

