# Supplementary material for: A Comparative Analysis of Bioactive Compounds and Heavy Metal Content in Table Eggs from Various Housing Systems
Source: Foods. 2026 Aug 14;15(16):2835. doi: 10.3390/foods15162835 (PMC13512252; doi:10.3390/foods15162835)
Supplement: Supplementary file 1 [file foods-15-02835-s001.zip › foods-4447760-supplementary.pdf]

Supplementary Materials

A Comparative Analysis of Bioactive Compounds and Heavy Metal Content in Table Eggs from Various Housing Systems

Kamil Drabik, Renata Zdun, Piotr Skałecki , Ewa Januś, Hanna Spasowska, and Justyna Batkowska

Table S1. The basic composition (%) of feed mixtures depending on bird's rearing system.

Feed mixture	Dry matter	Crude protein	Crude fat	Crude fiber	Crude ash
Organic (0)	91.76	10.56	5.06	4.55	12.31
Barn (1)/free range (2)	91.45	16.75	3.40	3.58	10.64
Cage (3)	92.15	16.19	6.57	5.88	14.45

Table S2. The fatty acids content in the samples feed mixtures (%).

Parametr	Organic (0)	Barn (1)/free-range (2)	Cage (3)
Saturated (SFA)			
C6:0	<LOD=0.016	0.02	0.07
C8:0	<LOD=0.016	<LOD=0.016	0.04
C10:0	<LOD=0.016	<LOD=0.016	<LOD=0.016
C11:0	<LOD=0.016	<LOD=0.016	0.02
C12:0	<LOD=0.016	0.04	0.03
C13:0	<LOD=0.016	<LOD=0.016	<LOD=0.016
C14:0	0.31	0.21	0.17
C15:0	0.07	0.05	0.09
C16:0	22.04	16.62	16.14
C17:0	0.16	0.14	0.15
C18:0	6.19	5.63	4.81
C20:0	0.08	0.24	0.48
C21:0	<LOD=0.016	<LOD=0.016	0.04
C22:0	0.09	0.25	0.56
C23:0	0.02	0.04	0.10
C24:0	0.06	0.13	0.28
Monounsaturated (MUFA)			
C14:1 n-5	0.06	0.02	0.05
C15:1 n-5	<LOD=0.016	<LOD=0.016	0.04
C16:1 n-7	2.42	1.08	0.26
C17:1 n-7	<LOD=0.016	<LOD=0.016	<LOD=0.016
C18:1 n-9 cis+trans	37.71	32.72	27.42
C20:1 n-15	<LOD=0.016	<LOD=0.016	<LOD=0.016
C20:1 n-9	0.36	0.22	0.47
C22:1 n-9	0.12	0.08	0.21
C24:1 n-9	<LOD=0.016	<LOD=0.016	0.04
Polyunsaturated (PUFA)			
C18:2 n-6c + C18:2 n-6t	23.60	36.46	40.17

C18:3 n-6 (γ)	0.05	0.04	<LOD=0.016
C18:3 n-3 (α)	1.11	2.16	3.21
C20:2 n-6	0.11	0.08	0.02
C20:3 n-6	0.07	0.04	<LOD=0.016
C20:4 n-6	0.94	0.54	<LOD=0.016
C20:3 n-3	<LOD=0.016	<LOD=0.016	<LOD=0.016
C20:5 n-3	<LOD=0.016	<LOD=0.016	<LOD=0.016
C22:2 n-6	0.04	0.03	0.03
C22:6 n-3	0.34	0.12	<LOD=0.016
Fatty acids indexes			
SFA	29.02	23.37	22.98
MUFA	40.67	34.12	28.49
PUFA	26.26	39.47	43.43
n-3 group	1.45	2.28	3.21
n-6 group	24.81	37.19	40.22
n-9 group	38.19	33.02	28.14

LOD – limit of detection; C6:0 – caproic acid, C8:0 – caprylic acid, C10:0 – capric acid, C11:0 – undecylic acid, C12:0 – lauric acid, C13:0 – tridecylic acid, C14:0 – myristic acid, C15:0 – pentadecylic acid, C16:0 – palmitic acid, C17:0 – margarit acid,, C18:0 – stearic acid, C20:0 – eicosanoic acid, C21:0 – heneicosanoic acid, C22:0 – behenic acid, C23:0 – tricosylicacid, C24:0 – lignoceric acid, C14:1 n5 – myristoleic acid, C15:1 n-5 – pentadecenoic acid, C16:1 n-7 – palmitoleic acid, C17:1 n-7 – heptadecenoic acid, C18:1 n-9 cis+trans – sum of oleic and elaidic acids, C20:1 n-15 – eicosenoic acid, C20:1 n-9 – gondoic acid, C22:1 n-9 – erucic acid, C24:1 n-9 – nervonic acid, C18:2 n-6c + C18:2 n-6t – sum of cis and trans linoleic acids, C18:3 n-6 (γ) – γ-linolenic acid (GLA), C18:3 n-3 (α) – α-linolenic acid (ALA), C20:2 n-6 – eicosadienoic acid, C20:3 n-6 – dihomo-γ-linolenic acid (DGLA), C20:4 n-6 – arachidonic acid (AA), C20:3 n-3 – eicosatrienoic acid (ETE), C20:5 n-3 – eicosapentaenoic acid (EPA), C22:2 n-6 – docosadienoic acid, C22:6 n-3 – docosahexaenoic acid (DHA); SFA – saturated fatty acids, MUFA – monounsaturated fatty acids, PUFA – polyunsaturated fatty acids.

Table S3. The heavy metals content [ppm] in the samples feed mixtures.

**Feed mixture**	Pb	Cd	As
Organic (0)	0.609	0.021	trace*
Barn (1)/free range (2)	0.196	0.019	trace
Cage (3)	0.450	trace	trace

*trace means value <LOQ (limit of quantification), Pb – lead, Cd – cadmium, As – arsenic.

Table S4. The proportion (%) of particular fatty acids in egg yolks depending on bird's rearing system.

Trait	The rearing system								F test (p-value)
	Organic (0)		Free-range (1)		Barn (2)		Cage (3)		
	$\bar{x}$	SD	$\bar{x}$	SD	$\bar{x}$	SD	$\bar{x}$	SD	
Saturated (SFA)									
C14:0	0.393[b]	0.040	0.293[a]	0.021	0.313[a]	0.010	0.312[a]	0.027	*0.000*
C15:0	0.067[b]	0.010	0.060[ab]	0.009	0.052[a]	0.009	0.053[a]	0.012	*0.002*
C16:0	24.12[b]	0.967	21.94[a]	0.471	22.66[a]	1.145	22.14[a]	0.853	*0.000*
C17:0	0.177[a]	0.026	0.187[b]	0.025	0.160[a]	0.012	0.160[a]	0.032	*0.028*
C18:0	7.840[b]	0.974	6.868[a]	0.616	7.147[ab]	0.526	7.155[ab]	0.345	*0.009*
C20:0	0.028[b]	0.012	0.020[a]	0.000	0.022[ab]	0.004	0.020[ab]	0.000	*0.008*
Monounsaturated (MUFA)									
C14:1 n-5	0.105[b]	0.023	0.057[a]	0.010	0.055[a]	0.010	0.055[a]	0.013	*<0.001*
C16:1 n-7	3.472[b]	0.439	2.457[a]	0.367	2.518[a]	0.273	2.548[a]	0.321	*<0.001*
C18:1 n-9	41.47[a]	1.727	42.50[a]	1.837	43.60[ab]	3.489	45.34[b]	2.188	*0.002*
C20:1 n-9	0.215[a]	0.048	0.268[c]	0.026	0.257[bc]	0.020	0.225[ab]	0.018	*<0.001*
Polyunsaturated (PUFA)									
C18:2 n-6	14.06[a]	1.947	18.40[b]	2.521	16.63[ab]	3.404	15.26[a]	2.332	*0.001*
C18:3 n-6 γ	0.082	0.012	0.085	0.010	0.090	0.023	0.082	0.023	*0.666*
C18:3 n-3 α	0.998[c]	0.213	0.488[b]	0.054	0.373[ab]	0.097	0.330[a]	0.063	*<0.001*
C20:2 n-6	0.138[a]	0.029	0.192[b]	0.046	0.160[ab]	0.052	0.123[a]	0.019	*<0.001*
C20:3 n-6	0.145[b]	0.041	0.143[b]	0.022	0.133[b]	0.025	0.103[a]	0.010	*0.001*
C20:4 n-6	1.408	0.140	1.397	0.075	1.325	0.117	1.267	0.209	*0.069*
C20:3 n-3	0.025[ab]	0.008	0.020[b]	0.000	-	-	-	-	*0.241*
C22:2 n-6	0.055[ab]	0.014	0.065[b]	0.013	0.047[a]	0.010	0.070[b]	0.022	*0.003*
C22:6 n-3	0.673[b]	0.073	0.340[a]	0.000	0.360[a]	0.023	0.360[b]	0.000	*<0.001*

[a, b] – means differ significantly at p≤0.05; SD – standard deviation; LOD – level of detection; C14:0 – myristic acid, C15:0 – pentadecylic acid, C16:0 – palmitic acid, C17:0 – margarit acid,, C18:0 – stearic acid, C20:0 – eicosanoic acid, C14:1 n5 – myristoleic acid, C16:1 n-7 – palmitoleic acid, C18:1 n-9 cis+trans – sum of oleic and elaidic acids, C20:1 n-9 – gondoic acid, C18:2 n-6 – linoleic acids, C18:3 n-6 (γ) – γ-linolenic acid (GLA), C18:3 n-3 (α) – α-linolenic acid (ALA), C20:2 n-6 – eicosadienoic acid, C20:3 n-6 – dihomo-γ-linolenic acid (DGLA), C20:4 n-6 – arachidonic acid (AA), C20:3 n-3 – eicosatrienoic acid (ETE), C20:5 n-3 – eicosapentaenoic acid (EPA), C22:2 n-6 – docosadienoic acid, C22:6 n-3 – docosahexaenoic acid (DHA); SFA – saturated fatty acids, MUFA – monounsaturated fatty acids, PUFA – polyunsaturated fatty acids.
